# Reports of Societies

**Published:** 1875-12

**Authors:** 


### Reports of Societies.

### CHICAGO MEDICAL SOCIETY.

*Regular Meeting, September* 27, 1875.

(Reported by D. C. STILLIANS, M.D.)

The President, Dr. Wm. E. Clarke, in the chair.

The report of the section on Obstetrics being called for, Dr. Wm. E. Quine presented the following cases:

In 1872, I saw, in consultation with Dr. Stillians, a multipara who imagined herself miscarrying at the seventh month. Excepting unusual rapidity of abdominal enlargement, nothing had occurred to attract her attention until about the fourth month, when without known cause, feeble pains and moderate hæmorrhage came on, and about a pint of aggregated "little bladders" was discharged from the womb. From the fourth to th seventh month the abdomen and mammæ had enlarged rapidly, but the patient was not sure that she had felt fœtal movements. From time to time, during periods of temporary malaise, small quantities of the cystic matter had come away; and a few days before the patient came under our observation, a midwife was summoned to attend her in labor.

A large quantity, estimated at over a quart, of the same kind of matter was discharged during the attendance of the midwife, who sent for Dr. Stillians. The abdomen was then about as large as at the seventh month of pregnancy. The patient was pale and anxious, but otherwise in good condition—the os being soft and patulous, and occupied by the cysts. The treatment consisted in the removal of the hydatidiform mass, and the subsequent use of ergot and injections of subsulphate of iron. The patient recovered.

It is the opinion of modern authors that the pathological changes in this disease occur in the chorion villi, and this of course involves the assumption that it is always the product of conception; but opinions are divided as to the precise manner in which these changes are brought to pass. The cysts are *not* true hydatids, and the latter are rarely found in the uterus. Paget holds that the primary departure from health occurs in the chorion, and is manifested in cystic enlargement of the cells of the villi. From the cells on the outer surface of each primary cyst new villi sprout, which, in turn, are converted into other cysts, and thus the process continues indefinitely. According to Virchow, each placental villus consists of an epithelial covering, and a matricular tissue, which is continuous with the proper mucous tissue of the umbilical cord.

In hydatidiform degeneration, there is proliferation of the investing epithelial cells and mucous degeneration of their contents; and the intercellular substance presents the characteristics of a myxomatous growth.

FRACTURES IN OPERATIVE MIDWIFERY.

In 1870, a primipara, in the County Hospital, was seized with violent antepartum convulsions during a tedious labor, with pelvic presentation. I hooked two fingers into the flexure of the thigh; made the necessary tractive force for delivery, and fractured the left femur. The limb was dressed at once, and for a few days the baby seemed well, but the nurse, without authority, removed the dressings in order to cleanse them, and the baby was immediately attacked with convulsions, of which it soon died.

In the early part of 1873, I was called to assist Dr. Stillians in a case of pelvic presentation, in which there was cessation of uterine contractions. The doctor's fingers were exhausted in a vain attempt to effect delivery. Under my manipulations, the baby was born with a broken thigh. A plaster of paris dressing was at once applied, and the patient did well. Some of the most authoritative writers of the day regard this accident as occasionally unavoidable. I could easily see how, with an equal amount of tractive force, *two fingers* in the flexure of the thigh would more readily fracture the shaft of the bone than a blunt hook; and I determined to employ the latter in future emergencies of the same kind; and last summer I had the felicity to deliver the child *without* fracturing the shaft of its femur—I only fractured the neck. It is not very uncommon in podalic version, especially when the liquor amnii has been long evacuated, and the uterus is firmly contracted upon its contents, to fracture arms and legs. A case of placenta prævia occurred in the practice of one of our most skillful accoucheurs, in which labor was induced by sponge tents and Barnes' dilators, and podalic version performed, immediately upon the evacuation of the liquor amnii. A "green stick" fracture of the humerus was produced, and the baby died soon after its birth.

### EXTRA UTERINE PREGNANCY.

In the latter part of 1869 a young primipara was admitted to the County Hospital, when Dr. H. W. Jones was attending accoucheur and myself the house physician. The patient was anæmic and emaciated to an extreme degree, and was far advanced in pulmonary phthisis. She stated on admission that she was eleven months pregnant, and that six months previously while in ordinary health she was suddenly seized with excruciating pain in the abdomen, faintness and vomiting. The physician in attendance pronounced the malady to be inflammation of the bowels, and under his treatment for this, the patient slowly regained a tolerable degree of comfort. She noticed upon recovery that the fœtus seemed to be lying loosely in the abdominal cavity; and she could distinctly feel its outlines, and even distinguish its hand from its foot. Up to the eighth month movements of the child continued strong. Her appetite was poor, bowels very loose, cough was troublesome and attended with profuse muco-purulent expectoration. An examination confirmed all that the patient had said about the apparent position, and freedom of the child from attachment. When she turned from one side to the other, the child would fall to the dependent part, and if she assumed the knee-elbow position the pelvis of the child could be felt in the epigastric region. The uterus was five and a half inches deep, and empty. Operative interference was put entirely out of the question, by the nature and advanced stage of the pulmonary disease. The patient died of exhaustion two weeks after admission.

Both lungs were found to be partially destroyed, and there was tubercular ulceration of the bowels. Old peritoneal adhesions indicated the pre-existence of inflammation. The product of conception lay in the left side of the false pelvis and abdomen, and was enclosed in what Dr. Jones ascertained to be the enormously dilated and thickened fallopian tube. The fœtus was not freed from its unnatural connections, and there was no discoverable rupture of the investing tissue. The contents of the sac were a dry, shriveled, but fully developed six-pound fœtus, and a moderate sized placenta, well advanced in fatty degeneration. There was no liquor amnii, and nothing in the peritoneal cavity to indicate that rupture of the tubular investment had taken place. The left ovary could not be found, and the fringed extremity of the tube was lost upon the expansion. The uterus was large, and its mucous membrane thickened. The suspicion may be entertained that when the patient had the so-called attack of *enteritis*, there was really rupture of the sac and escape of its fluid contents into the peritoneal cavity, giving rise to inflammation, and subsequent absorption of the liquor amnii.

PLACENTA PRÆVIA.

A young primipara had nearly arrived at full term, and was extremely anæmic from repeated hæmorrhages. The last of these had commenced two days before I saw her, and a physician had been summoned, who had resorted to the internal use of ergot, acetate of lead and the tampon. On my arrival the patient was having feeble labor pains. She was very pale and weak, apprehensive, sick at stomach, and her pulse was rapid, small and soft. Upon the removal of a quantity of cotton saturated with solution of subsulphate of iron, from the vagina, I found a soft dilatable os, slightly encroached upon by left lateral implantation of the placenta, whence a pretty free stream of blood issued. The vertex presented, forceps were at once applied, and during the extraction of the child a full dose of ergot was administered. Quinine and brandy were subsequently used and the patient recovered, though her infant was born dead.

A second case occurred last winter, in the practice of Dr. Adolphus. A primipara had hæmorrhage early in pregnancy. Subsequently the hæmorrhages were not very frequent nor alarmingly profuse, and toward the conclusion of gestation, when it was deemed advisable to induce labor, the patient was still in good condition. Sponge tents and Barnes' dilators were employed for dilatation, and a cross presentation was discovered; podalic version was employed, and delivery with forceps. The patient lost little if any more blood than she would in natural labor. The child, though born living, had a "green stick" fracture of the humerus, and died on the following day. The mother seemed to bear the operation well. A pretty free hæmorrhage occurring after the expulsion of the placenta, it was deemed advisable by the attendant to inject a diluted solution of sulphate of iron into the uterus. This was done, and ergot was also given internally. I

did not see the patient after this time, but was informed that she died in a day or two of puerperal fever.

In a case such as that just described, and in flooding from other causes, the local use of astringents is worse than useless, although it is approved by many writers.

Dr. E. Fletcher Ingals reported the following case:

### CONGENITAL ABSENCE OF ONE KIDNEY.

Though it is by no means novel at post-mortem examinations to find but one kidney, yet these cases are perhaps of sufficient rarity to render the notes which I have kept of one, of interest to this Society. The subject in which this abnormal condition was found was a coroner's case that was brought to the city morgue, in July, 1872. The post-mortem was made by Dr. Miller, in presence of Drs. Strong, Chenoweth and myself. Parties who had known the deceased, stated that he had a habit of almost constant intoxication; and it was ascertained that he had frequently applied at the dispensary for treatment on account of dropsy. The abdominal and thoracic organs were loaded with fat; aside from this, the heart, lungs and spleen appeared normal. The liver was but slightly altered in size, of a yellowish tinge and somewhat softened. The left kidney, to the eye, seemed of normal size; it was paler than natural and presented a roughened surface. Bisecting the kidney we observed two cysts, one about the size of a filbert and the other the size of a large pea. The renal tissue was pale and hard; the cortical substance thick, and the pyramids mostly obliterated. Not a trace of the right kidney could be found. The left ureter was somewhat enlarged. The lower end of the right ureter was found entering the bladder at the usual situation, from which point it rapidly decreased in size as it ascended toward the region of the kidney, and apparently came to an end about four inches from the bladder. This ureter was perforated by a canal about half a line in diameter.

In this case, the kidney was doubtless congenitally absent, but notwithstanding this and the abuse heaped upon the left kidney and the liver, the subject had lived to middle life.

---

### CHICAGO MEDICAL SOCIETY.

*Regular Meeting, Oct.* 4, 1875.

(Reported by D. C. Stillians, M.D.)

The President, Dr. Wm. E. Clarke, in the Chair.

Dr. J. S. Knox, from the committee on obstetrics, read a report on the subject of anæsthetics in labor.

Dr. Mary H. Thompson reported two cases, as follows:

### TWO SUBPERITONEAL TUMORS OF THE UTERUS.

Mrs. E. L. D. S., æt. 39; was married, but had separated from her husband several years ago. She is a woman of medium height, slender, with black eyes and hair; is rather pale and tremulous. She has been sick for three years.

Examination revealed two subperitoneal tumors of the uterus. One was nearly or quite sessile, and situated upon the upper and right side of the organ, and was about the size of a large orange. The second was found floating loose in the abdominal cavity. It was quite as large as a child's head at term, oblong and hard, and quite mobile. When the patient was lying on one side it would sometimes *ascend* to the other, or it would, while the patient was sitting, perhaps rise so as to press against the stomach. After taking from twenty to thirty drops of the fluid extract of ergot the tumor would become fastened in the brim of the pelvis with such force we were obliged to omit ergot for some time to give the patient rest.

History: Menses were always natural until the last epoch, which occurred a week too soon. She had been married twenty years, and had been pregnant but once, and that about fourteen years ago. She then had a miscarriage at five months, and did not feel well for a long time after. Eight years ago she fell and bruised herself across the lower part of the abdomen, against a stone. She never noticed much pain there, after the first soreness had passed away, until three years ago, when she found a lump as large as a teacup. She never had had leucorrhœa.

The peduncle of the floating tumor could not be perceived when the patient was under the influence of ergot. Ergot was given by the advice of Dr. Byford, to kill the growth, but, causing so much pain, we omitted its use for more than a week, until the growth began to move upward in the abdominal cavity.

### HÆMATOCELE AND RUPTURED PERINEUM.

Mrs. F. S.; American; æt. 23; a primipara; entered hospital in labor Sept. 15th, at 10.30 A. M., when the first stage was nearly completed. When the head began to enter the pelvis the pains became violent, and the labor was completed in less than one hour. In about an hour after delivery the patient complained of great pain, which seemed of a different character from "after pains." A tumor was then discovered bulging into the right labium, which did not extend up very high into the vagina. Soon after 3 P. M. the patient had continuous pain of an intensity increased periodically, but at shorter intervals than "after pains," and also pain in the rectum.

The hard tumor was found to be more prominent in the right side of the vagina than elsewhere, although it also involved the right labium and the perineum, and distended the latter with every pain, like a child's head.

The tumor increased in size, and soon proved to be an hæmatocele. Pressure occasioned so much pain that it was discontinued. After a time there was considerable flowing. The tumor was now smaller and filled with clots, and an opening occurred about two inches above the ostium vaginæ, large enough to permit the introduction of three fingers. To check further hæmorrhage, I applied a tampon, which was not removed until morning.

The catheter was used twice daily for a week, when, spasm of the urethra occurring, I ordered extract. belladonnæ fluid., in three-drop doses, till the sphincter yielded to its influence. The vagina and the sac of the hæmatocele were washed twice daily for six or eight days with a solution of carbolic acid. Six days after labor the opening would admit the tip of one finger only, and was but an inch from the orifice of the vagina, and thirteen days after it was entirely closed. The general pelvic tissue was somewhat hardened at this date, but this was fast wearing away. The perineum was ruptured to the sphincter ani.

---

### CHICAGO MEDICAL SOCIETY.

*Regular Meeting October* 18, 1875.

[Reported by CHAS. WARRINGTON EARLE, M.D.]

The President, Dr. Wm. E. Clarke, in the chair.

Prof. H. M. Lyman read a paper on Infantile Hæmorrhage. [Printed in the JOURNAL AND EXAMINER for November].

Dr. Rosa H. Engert remarked that the disease under consideration, fortunately, was very rare. Vogel, in his work, mentioned only two in 1,000 cases. She believed it to be a disease of the blood—a dyscrasia. Two or three mild cases had occurred in her practice—all recovered.

Dr. A. H. Foster related the history of a case happening in the family of a neighboring practitioner. When at the age of about two days, the child began to have bloody passages, these being repeated about every half hour. Cold applications were made to the abdomen, and injections of plumb. acet. administered. The hæmorrhage continuing, Dr. Skeer was called in, and suggested injections of alum. During the night following, the bloody passages commenced again, and the fld. ext. hamamelis (about ʒj) was given by rectum. No more hæmorrhage occurred.

Dr. Paoli said that the etiology of this severe difficulty was very obscure, and he had been able in but a single case to find a sufficient cause for the generally fatal result. At the autopsy of the case referred to, he found complete obliteration of the ductus communis choledochus. These hæmorrhages take place during and after a number of diseases, also where the nutrition is insufficient. In Norway where the people live principally on fish, many cases had been noticed.

Dr. Van Buren related the history of a case in which there was a great tendency to hæmorrhage at the umbilicus. It was evidently from a hæmorrhagic diathesis. A second case occurring in his practice, had a protrusion over the posterior fontanelle, which he supposed to be sanguineous. The child died very suddenly a few days after it was noticed.

Dr. Earle had never seen a case, but called attention to a class of cases not necessarily associated with purpura hæmorrhagica. It was probable, according to Dr. J. Lewis Smith, that too speedy ligature of the cord may have something to do with the production of this kind of hæmorrhage.

---

### CHICAGO SOCIETY OF PHYSICIANS AND SURGEONS.

*Regular Meeting, Oct.* 25, 1875.

[Reported by E. Warren Sawyer, M.D.]

President, Dr. Bevan, in the chair.

Dr. Merriman, the chairman of the Section on Obstetrics and Diseases of Women and Children, read a paper on puerperal fever; which was a review and examination of the recent discussion "On the relation of puerperal fever to the infective diseases and pyæmia," by the Obstetrical Society of London. After relating how the subject of discussion had been divided into six chief questions, by Spencer Wells, Dr. Merriman reviewed succinctly, and in order, the remarks of each discusser.

In conclusion, the reader thought the results of the examination by the London Society, warranted him in giving a definite answer to the first three questions asked by Mr. Wells:

1. Is there any form of continued fever, communicated by contagion, and occurring in connection with childbirth, which is distinctly caused by a special morbid poison, and as definite in its progress and the local lesions associated with it, as typhus or typhoid, scarlet fever, measles, or small-pox?

To this, the reader thought the weight of evidence enabled him to answer no.

2. May all forms of puerperal fever be referred to attacks of some infective fever—as scarlet fever, or measles—occurring in connection with childbirth, on the one hand; or, on the other, to some form of surgical fever, or to erysipelas, caused by or associated with changes in the uterus and neighboring parts following the process of childbirth?

This question could be answered affirmatively.

3. If all cases of contagious and infectious diseases which occur under other conditions than at childbirth are set aside, does there remain any such disease as puerperal fever?

Dr. Merriman thought the majority of the gentlemen of the Obstetrical Society had answered this in the negative.

In the discussion of Dr. Merriman's paper, Dr. Byford said that, had he taken part in the discussion of this question, he would have drawn particular attention to the contrast between hospital and private practice. He thought that the majority of the adherents of the theory of the contagiousness of puerperal fever, founded their conclusions upon hospital practice; there is a chance of error in this. He had witnessed most destructive epidemics of so-called puerperal fever even in private practice; but this, he thought, was not because of the contagiousness of the disease, *per se*, but on account of a condition of the atmosphere, favorable to the extension of an epidemic. This condition is constant in hospitals, to that degree that the word hospitalism has been given to it. He did not believe that the so-called puerperal fever is contagious; and, on the whole, acceded to the conclusions drawn by Dr. Merriman.

Dr. Simon said that, during a recent endemic of scarlet fever, he had, in the midst of the disease, delivered a woman who was not, in the least, made worse by her surroundings.

The President remarked that some years ago he attended a woman through labor, who, at that time, had the bronchitis and premonitory fever of measles, but the woman had no puerperal trouble; though the child took measles. Frequently, while in attendance at the County Hospital through epidemics of puerperal fever, he had attended confinement cases in private practice, and once his own wife, without harm to his patients. He always observed the precaution of washing his hands in disinfectants.

Dr. C. Gilman Smith said that he once delivered a woman who had facial erysipelas, but the woman had no puerperal complication.

Dr. Bartlett was once called to a woman who was miscarrying at the sixth month, and who then had the eruption of measles at its height; twice he was obliged to empty the uterus for internal post-partum flowing; notwithstanding all this, the convalescence of the patient was not interrupted. Years ago he made frequent dissections and autopsies, and his personal uncleanness "stank to heaven" and was notorious, but he was not deterred from attending upon confinement cases without harm.

Dr. Sawyer read a paper on the second stage of labor, and exhibited the new obstetric forceps of Newman; also his modifications of the same.

In the discussion of the paper, Dr. Bartlett said, that drawing the fœtal head through the cervix uteri would sometimes tear the organ, for he had witnessed the accident. He had no doubt that the forceps might be used oftener with benefit; but, before resorting to this instrument, we should try other aids to the uterine contractions, which are less dangerous than ergot, as coffee, cinnamon, quinine, cimicifuga, borax, etc. Sometimes a change of position is a help; he had found that the kneeling position favored the uterine contractions.

Dr. Merriman said that he had increased the force of the uterine contractions by six grains of quinine.

Dr. Owens inquired if quinine was to be relied upon to increase or to arouse the force of the uterus; and if it cannot always be given to a pregnant woman.

Dr. Hyde said it is now agreed that quinine is not an oxytocic, nor an emmenagogue, but that it sometimes increased the uterine force, which was already aroused, by its general stimulant effect.

Dr. Byford remarked that he gave large doses of quinine fearlessly to pregnant women. In referring to the paper, he said that there was another great danger to the fœtus in giving ergot to the woman in labor, viz.: the toxic effect of the drug, which was sufficient to destroy the fœtus even when labor was almost completed. In speaking of the new forceps, Dr. Byford said that the instrument was an important aid; he thought Dr. Sawyer's modification a good one, and were he now in the active practice of obstetrics, he would carry this instrument in his pocket to every case of confinement.

Dr. Smith thought the forceps could be improved by widening the blade and the fenestra.

After the discussion of the foregoing paper, Dr. Smith exhibited Dr. Jones's bi-valve vaginal speculum. The handle of the instrument is upon the right side of the speculum, and, when introduced, rests against the internal surface of the patient's right thigh. It is so constructed, further, that the opening of the blades does not stretch the ostium vaginæ. Dr. Smith also exhibited a specimen of vaseline, which he had recently used for lubricating bougies and specula.

